# HLA-F*01:01 presents peptides with N-terminal flexibility and a preferred length of 16 residues

**DOI:** 10.1007/s00251-019-01112-1

**Published:** 2019-04-02

**Authors:** Gia-Gia T. Hò, Funmilola J. Heinen, Trevor Huyton, Rainer Blasczyk, Christina Bade-Döding

**Affiliations:** 1 Institute for Transfusion Medicine, Hannover Medical School, Carl-Neuberg-Str. 1, 30625 Hannover, Germany; 2 Department of Cellular Logistics, Max Planck Institute for Biophysical Chemistry, Am Fassberg 11, 37077 Göttingen, Germany

**Keywords:** HLA-F, Peptides, pHLA-F model, Proteom

## Abstract

HLA-F belongs to the non-classical HLA-Ib molecules with a marginal polymorphic nature and tissue-restricted distribution. HLA-F is a ligand of the NK cell receptor KIR3DS1, whose activation initiates an antiviral downstream immune response and lead to delayed disease progression of HIV-1. During the time course of HIV infection, the expression of HLA-F is upregulated while its interaction with KIR3DS1 is diminished. Understanding HLA-F peptide selection and presentation is essential to a comprehensive understanding of this dynamic immune response and the molecules function. In this study, we were able to recover stable pHLA-F*01:01 complexes and analyze the characteristics of peptides naturally presented by HLA-F. These HLA-F-restricted peptides exhibit a non-canonical length without a defined N-terminal anchor. The peptide characteristics lead to a unique presentation profile and influence the stability of the protein. Furthermore, we demonstrate that almost all source proteins of HLA-F-restricted peptides are described to interact with HIV proteins. Understanding the balance switch between HLA-Ia and HLA-F expression and peptide selection will support to understand the role of HLA-F in viral pathogenesis.

**Electronic supplementary material:**

The online version of this article (10.1007/s00251-019-01112-1) contains supplementary material, which is available to authorized users.

## Introduction

Human leukocyte antigens (HLA) are key regulators of the immune system that scan the intracellular proteome and present self- or non-self-peptides to cells of the immune system (Klein and Sato 2000a). HLA molecules therefore constitute highly specific ligands for T cell receptor, since every single bound peptide alters the accessible surface for immune recognition of effector cells. The exclusive interactions between a T cell receptor and a peptide-HLA-Ia (HLA-A, HLA-B and HLA-C) complex are crucial to maintain the surveillance of cellular health (Zinkernagel and Doherty 1974). The outstanding polymorphic variability of HLA-Ia molecules enables the immune system to overcome the majority of pathogenic episodes (Klein and Sato 2000a; Klein and Sato 2000b). While HLA-Ia molecules are constitutively expressed on the cell surface, HLA-Ib molecules (HLA-E, HLA-F and HLA-G) are tissue specific, marginal polymorphic and their expression is triggered by distinct pathogenic conditions (Strong et al. 2003; Kraemer et al. 2014; Foroni et al. 2014). HLA-Ib molecules are involved in a diverse range of functions in adaptive and innate immunity. In recent years, knowledge about the function (Wagner et al. 2017; Kraemer et al. 2015; Celik et al. 2018a) and biophysical features (Celik et al. 2018b; Celik et al. 2016; Petrie et al. 2008) of HLA-Ib molecules are engaged in the fundamental understanding of tumor immunosurveillance, pathogen recognition, regulation of autoimmunity and virus-induced immunopathology (Kochan et al. 2013; Rebmann et al. 2014; Hofstetter et al. 2011), their upregulation during pathogenic episodes support the mediation of immune tolerance (Celik et al. 2018b). During viral infection, HLA-E is upregulated on the cell surface and avoids the recognition of infected HLA-Ia empty cells by NK cell (Kraemer et al. 2014). The presence of HLA-G is associated with immunosuppressive abilities. Consequently, expression of HLA-G is part of immune evasion strategies of many tumors to avoid immunosurveillance by T and NK lymphocytes (Morandi et al. 2016). HLA-F is the most enigmatic HLA-Ib molecule and its function has still not yet been comprehensively investigated. The genomic architecture of HLA-F is composed of seven exons. An internal stop codon within exon 6 leads to a truncated heavy chain through exclusion of exon 7 from the mature mRNA transcript (Geraghty et al. 1987; Geraghty et al. 1990). To date, 30 HLA-F alleles encoding for five proteins (HLA-F*01:01, HLA-F*01:02, HLA-F*01:03, HLA-F*01:04, and HLA-F*01:05) have been described (Robinson et al. 2015), HLA-F*01:01 as the predominant allelic variant (Pan et al. 2013; Manvailer et al. 2014). The HLA-F expression pattern is highly specific. HLA-F is mainly expressed in leukocytes, including monocytes, B cells, T cells, and NK cells (Lee et al. 2010). Additionally, HLA-F has been detected in non-small-cell-lung cancer (Lin et al. 2011), in esophageal squamous cell carcinoma (Zhang et al. 2013), gastric adenocarcinoma (Ishigami et al. 2013), and breast cancer (Harada et al. 2015).

HLA-F is also upregulated on the surface of HIV-infected CD4^+^ T cells (Garcia-Beltran et al. 2016) where the interaction between HLA-F and the NK cell receptor KIR3DS1 triggers an antiviral cytokine response leading to the inhibition of HIV-1 replication. Although many viruses have developed mechanisms to avoid HLA expression during infection (Mwimanzi et al. 2012), HLA-F expression is upregulated during HIV infection while its interaction with KIR3DS1 is diminished (Garcia-Beltran et al. 2016).

While the function and peptide features of the HLA-Ib molecules HLA-G and HLA-E are well characterized (Kraemer et al. 2015; Celik et al. 2018a), HLA-F has not yet been comprehensively analyzed. Recent studies have reported HLA-F to be presented on the cell surface either with or without peptide (Goodridge et al. 2013a; Dulberger et al. 2017). Peptide-empty HLA-F molecules function as open conformer (OC) that binds to HLA class I molecules (Goodridge et al. 2013a; Goodridge et al. 2013b). Furthermore, there is evidence that the OC form of HLA-F is a ligand for NK cell receptors (Garcia-Beltran et al. 2016; Goodridge et al. 2013b; Burian et al. 2016). The peptide bound form of HLA-F presents peptides with classical HLA-Ia features (Dulberger et al. 2017). Usually, the pool of presentable peptides has to pass several selection criteria, such as the individual HLA allele-specific peptide binding motif before being presented by an individual HLA molecule. Before being presented by an HLA-Ib molecule, the selection criteria are understandably more severe due to the limited polymorphisms in HLA-Ib molecules (Braud et al. 1997). While peptide/HLA-E expression levels are highly dependent on cellular stress (van Hall et al. 2010; Sasaki et al. 2016), HLA-G selects tissue-specific peptides (Celik et al. 2018b) that are independent of peptide anchor motifs (Celik et al. 2018a). The restricted interaction between peptide/HLA-F (pHLA-F) complexes and the NK cell receptor KIR3DS1 during HIV infection suggests that understanding HLA-F peptide selection and presentation is functionally important. Despite this, the full biological peptide selection criteria of HLA-F have not yet been thoroughly investigated especially in infection situations.

We therefore hypothesized that HIV might trigger an altered peptide selection that results in the presentation of a newly selected set of peptides by HLA-F, the availability of certain pHLA-F complexes might modify the docking mode of KIR3DS1.

The first step for the functional understanding of HLA-F is to analyze the characteristics of the particular peptides and the peptide repertoire. The knowledge of the peptide features will guide towards understanding the molecules structure and function.

## Material and methods

### Cell lines

All cell lines were maintained at 37 °C and 5% CO_2_. HLA class I negative *LCL721.221* cell lines transduced with sHLA-F*01:01 (soluble, exons 1–4) were maintained in RPMI1640 (Lonza, Basel, Switzerland) supplemented with 10% heat-inactivated FCS (Lonza, Basel, Switzerland), 2 mM L-glutamine (c. c. pro, Oberdorla, Germany), 100 U/ml penicillin, and 100 μg/ml streptomycin (c. c. pro, Oberdorla, Germany). The human embryonal kidney cell line *HEK293T*, used for production of lentiviral particles, was cultured in DMEM (Lonza, Basel, Switzerland) supplemented with 10% heat-inactivated FCS, 2 mM L-glutamine, 100 U/ml penicillin, 100 μg/ml streptomycin, and 1 mg/ml Geneticin® (Life Technologies, Carlsbad, USA).

### Large-scale production of sHLA-F*01:01

Constructs encoding for soluble HLA-F (sHLA-F*01:01, exons 1–4) were generated from *HEK293T* cDNA via PCR. The sequence for soluble HLA-F*01:01 along with an N-terminal V5-His6 tag was cloned into the lentiviral vector pRRL.PPT.SFFV.mcs.pre and verified through sequencing. According to the method described by Bade-Doeding et al. (Bade-Doeding et al. 2011), *HEK293T* cells were transfected with the target plasmid along with the packaging and envelope vectors psPAX2 and pmD2G. *LCL721.221* cells were stably transduced; the expression of trimeric sHLA-F*01:01 molecules was confirmed by ELISA as previously described (Celik et al. 2018b).

Large scale production of recombinant sHLA-F*01:01 was performed by soluble HLA technology (Kunze-Schumacher et al. 2014) using *CELLine* bioreactors (Integra Biosciences, Biebertal, Germany). Cell culture supernatant containing sHLA-F*01:01 molecules was harvested weekly and centrifuged to remove cells followed by filtration through a 0.45-μM membrane (Millipore, Schwalbach, Germany). Peptide bound sHLA-F*01:01 molecules were purified using an NHS-activated HiTrap column (Life Technologies, Carlsbad, USA) coupled to the mAb W6/32. Purified protein was quantitatively and qualitatively analyzed utilizing SDS gel electrophoresis and ELISA.

### Mass spectrometric analysis of the sHLA-F*01:01-bound peptides and the cell proteome

Peptides were eluted from purified sHLA-F*01:01 complexes by adding trifluoric acid (TFA, J. T. Baker, Phillipsburg, USA) at a final concentration of 0.1%. An Amicon Ultra centrifugal tube (Millipore, Schwalbach, Germany) with a 10-kDa cutoff membrane was used to separate peptides from the HLA molecules. The peptide fractions were purified using ZipTips (0.6 μl C18 resin, Merck, Darmstadt, Germany) and 50% acetonitrile/0.1% TFA for elution. The LC/MS analysis was performed with a Dionex Ultimate 3000 high-performance LC system and a LTQ Orbitrap Lumos mass spectrometer (Thermo Fisher, Waltham, USA). Peptide data were analyzed using the protein alignment tool BLAST and UniProt database. To examine the complete *LCL721.221* proteome, cells were lysed using RIPA buffer; the cell suspension was thoroughly vortexed and incubated on ice for 30 min. Following centrifugation (15 min, 13,000 rpm, 4 °C), the protein concentration was estimated using BCA protein assay kit (Thermo Fisher, Waltham, USA). Fifty microgram of protein was incubated at 95 °C for 5 min, alkylated by adding 1 μL 40% acrylamide, and separated using SDS gel electrophoresis. The lanes were cut into fractions, dehydrated with acetonitrile, and dried via Speedvac (Thermo Fischer, Rockford, USA). Protein digestion was performed with trypsin o/n. After an additional rehydration step, dried peptides were solved in 30 μL 2% acetonitrile/0.1% TFA for MS analysis. The LC/MS analysis was performed using a high-performance LC system and a LTQ Orbitrap Velos mass spectrometer (Thermo Fisher, Waltham, USA).

### Modeling of HLA-F

The HLA-F 01:01 structure (5KNM) from the Protein Data Bank (PDB) was taken for structure analysis. The software *pdbset* (CCP4) was used to remove anisotropic B-factors, water, NAG, and other chains leaving only the HLA-F*01:01 heavy chain and the peptide chain. *Pymol* was used to generate linear peptides. Linear peptides were superposed on C-terminal 3 residues of 5KNM peptide in program *coot* (CCP4) using lsq superpose routine. 5KNM peptide was removed and the peptides AVFVDLEPTVIDEVR and mVNPTVFFDIAVDGEPLGR were merged with heavy chain to create starting models for *rosetta* input. Rosetta FlexPepDock was used for peptide docking on the HLA-F model. The models were created and ranked based on their Rosetta generic full atom energy score.

## Results

### Characteristics of sHLA-F*01:01-restricted peptides

**Fig. 1:**
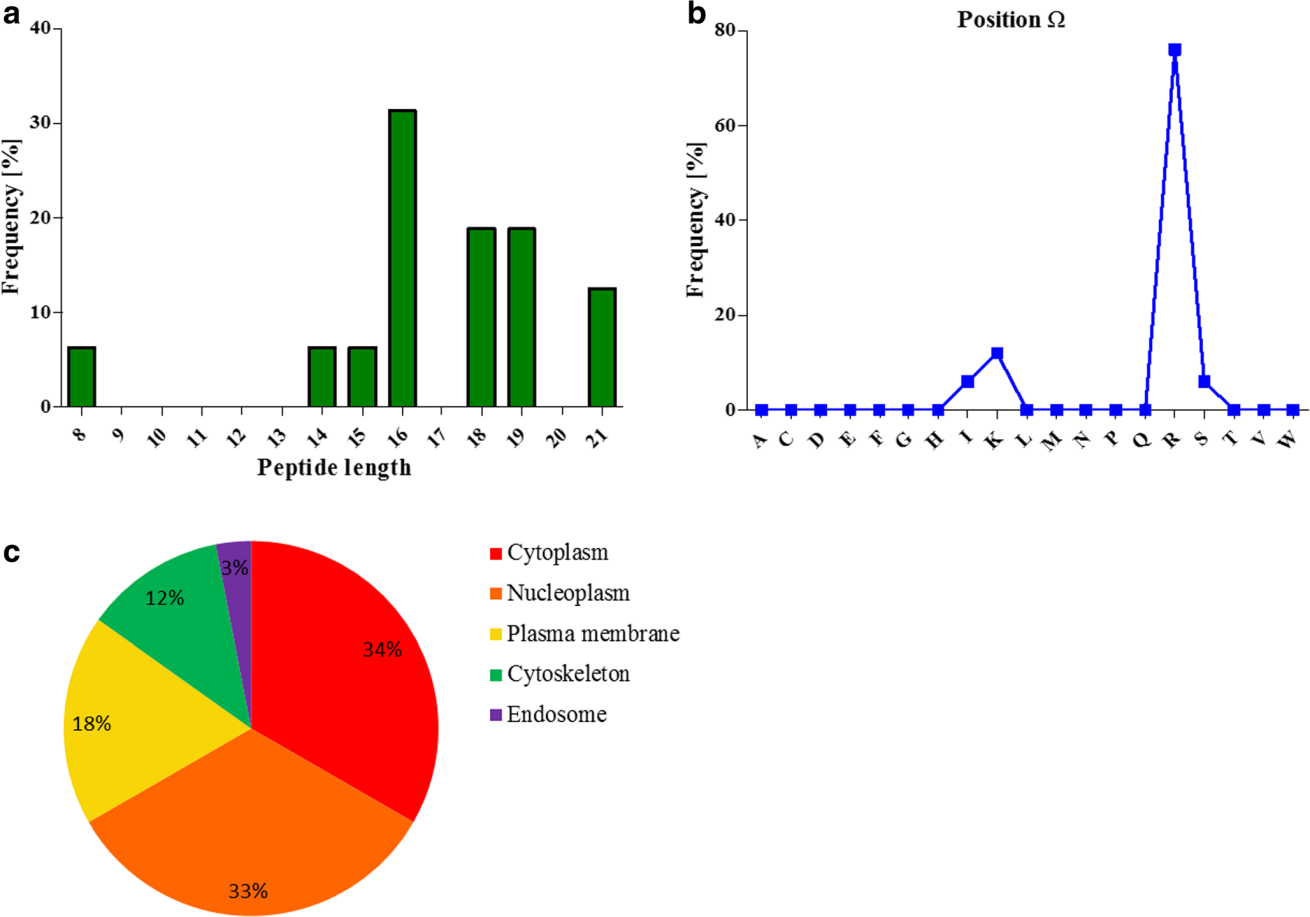
Characteristics of peptides presented by HLA-F*01:01. (**a**) Length distribution of peptides presented by sHLA-F*01:01. The majority of peptides exhibit a length of 16 to 19 AAs. The peptide length is given on the x-axis, percentage of observed peptides on the y-axis. (**b**) Frequency of AA at peptide position Ω. The respective AA is given on the x-axis, percentage of observed AA on the y-axis. (**c**) Cellular localization of the HLA-F*01:01 restricted peptide origin. HLA-F selects preferentially peptides of nucleoplasmatic or cytoplasmatic origin

sHLA-F*01:01 molecules were produced in *LCL721.221* cells and following affinity purification of trimeric sHLA-F complexes (Fig. S1), bound peptides were recovered and mass spectrometrically analyzed (Fig. S2). From 0.5 mg protein, 16 specific peptides were recovered (Table 1, Fig. 1).

**Table 1:** HLA-F*01:01-restricted peptides derived from *LCL721.221* cells

Sequence	Length	Protein origin	Gene name
wnSWDPRR	8	Proline-rich coiled-coil 2B	PRR2B
LVINGNPITIFQER	14	Glyceraldehyde-3-phosphate dehydrogenase	GAPDH
AVFVDLEPTVIDEVR	15	Tubulin alpha-1B chain	TUBA1B
LFIGGLSFETTDESLR	16	Heterogeneous nuclear ribonucleoprotein A1	HNRNPA1
SYELPDGQVITIGNER	16	Histamine receptor H3 subunit peptide 4	HRH3
GLGTDEDTLIEILASR	16	Annexin A1	ANXA1
FGVEQDVDmVFASFIR	16	Pyruvate kinase M	PKM
qGQSSIAmmGqGSQGS	16	Synovial sarcoma translocation gene on chromosome 18-like 1	SS18
VNPTVFFDIAVDGEPLGR	18	Peptidyl-prolyl cis-trans isomerase A	PPIA
AAVPSGASTGIYEALELR	18	Beta-enolase isoform X1	ENO3
vNPTVFFDIAVDGEPLGR	18	Peptidyl-prolyl cis-trans isomerase A	PPIA
TAFDEAIAELDTLSEESYK	19	YWHAZ protein	YWHAZ
mVNPTVFFDIAVDGEPLGR	19	Peptidyl-prolyl cis-trans isomerase A	PPIA
KPEQQGVmcVIEkTVDGqI	19	Usherin	USH2A
DLYANTVLSGGTTmYPGIADR	21	Beta-actin	ACTB
FDGALNVDLTEFQTNLVPYPR	21	Tubulin alpha-1B chain	TUBA1B

HLA-F*01:01 restricted peptides acquired in *LCL721.221* cells. Post translational modifications are marked by small letters. (m = oxidized; n = deamidated; q = deamidated, c = propionamide; k = acetylated; N-terminal small latter = N-term acetylated)

**Fig. 2:**
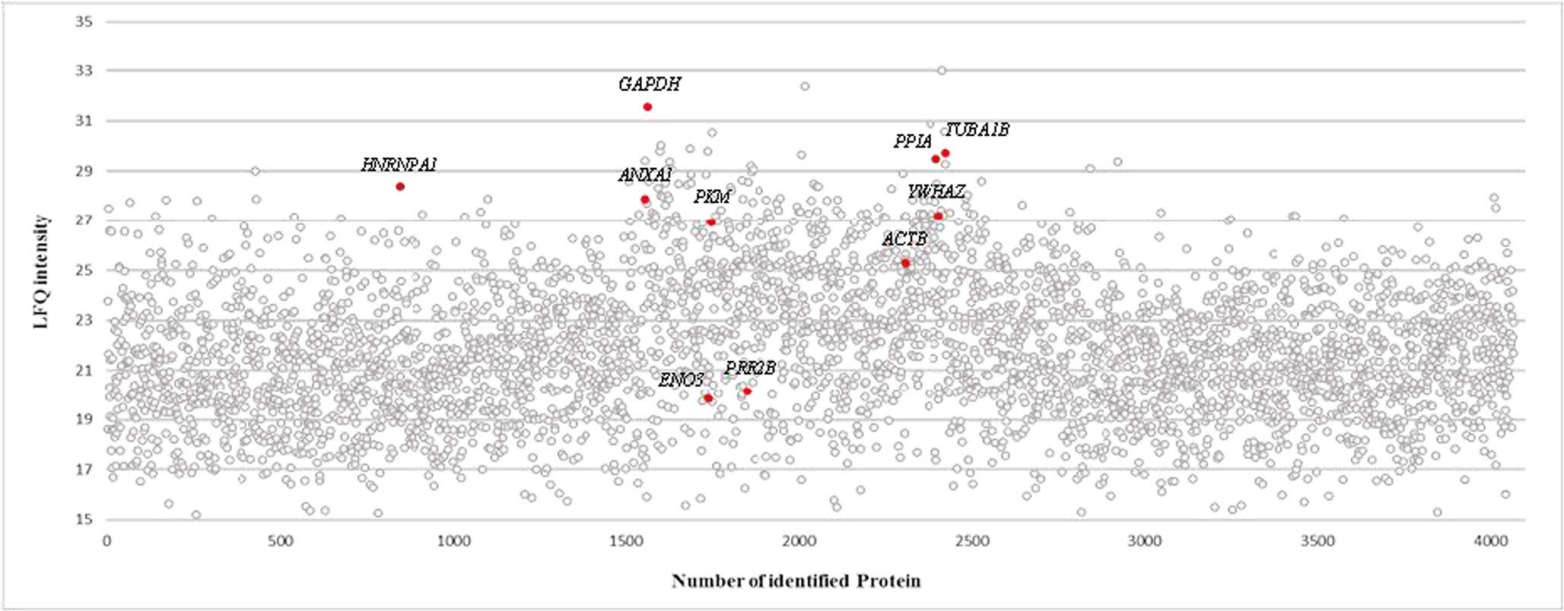
Protein plot of total proteome analysis from *LCL721.221* cells. 4067 proteins were detected (indicated as grey dots). 10 of the peptide source proteins could be verify by proteome analysis (indicated as red dots). Number of identified protein is given on the x-axis, the label free quantification (LFQ) intensity is given on the y-axis

In order to verify the biological occurrence of the source proteins, proteome analysis of *LCL721.221* cells was performed. Ten peptide source proteins could be confirmed by full proteome analysis (Fig. 2), while 3 peptide source proteins could not be detected by full proteome analysis. The peptides presented by HLA-F*01:01 exhibit a non-canonical length from 8 to 21 AAs (Fig. 1a), 16-mer peptides were found most frequently. Peptides are anchored by Arg (76%) at pΩ (Fig. 1b), while an anchor at p2 could not be identified. The peptides presented by HLA-F*01:01 are mainly derived from the cytoplasm and nucleoplasm (Fig. 1c).

### The majority of the proteins derived from peptides presented by HLA-F are described to interact with HIV-I proteins

To understand the relation of HLA-F-restricted peptides and HIV, we analyzed the protein source of HLA-F*01:01-restricted peptides for their described interaction with HIV-1 proteins (Table S1). Eight of the 13 source proteins are described as interaction partners of at least one HIV-1 protein (Table S1).

### The N-terminal site of the peptides is sticking out of the peptide binding pocket

**Fig. 3:**
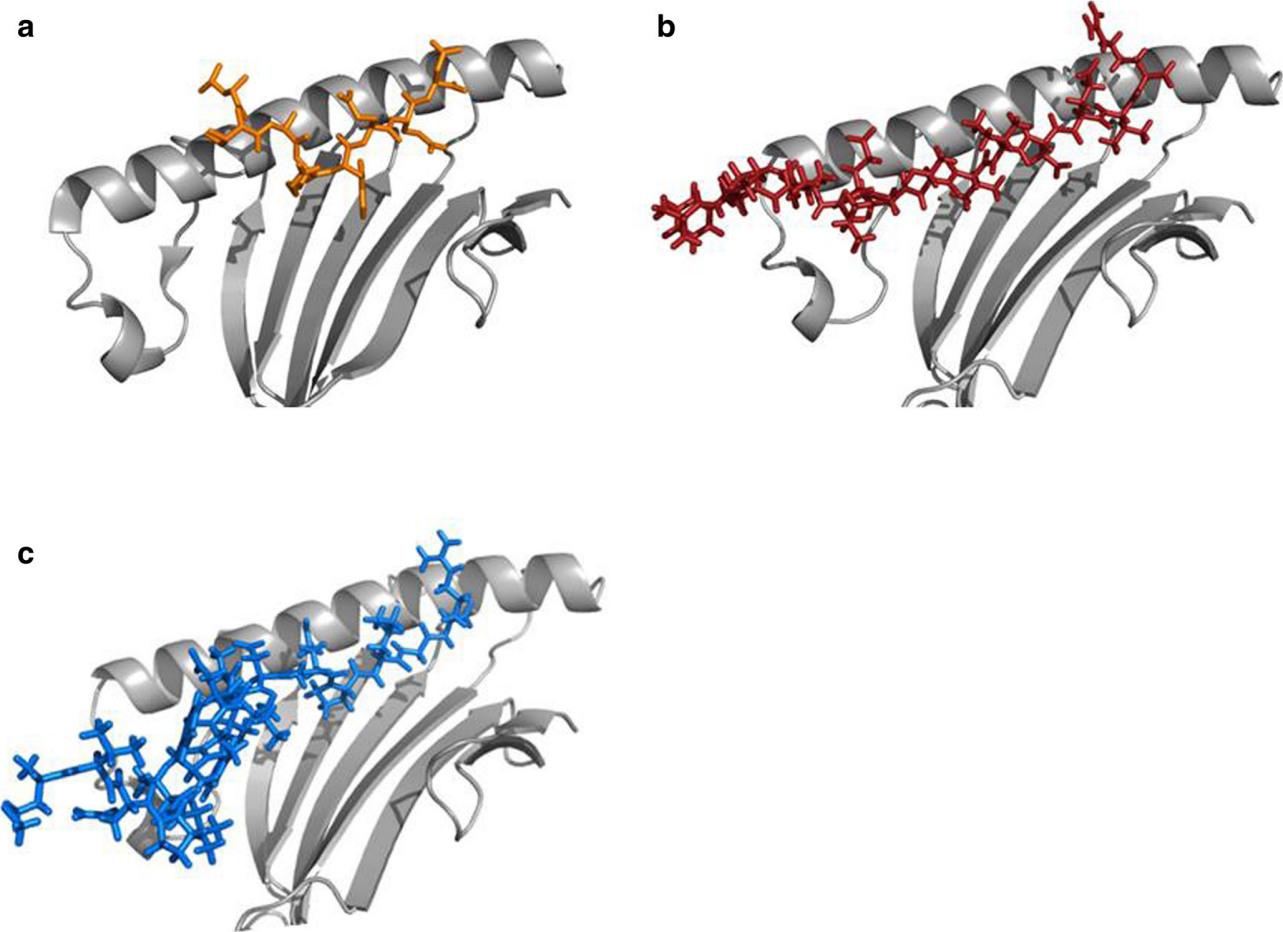
Structural comparison of pHLA-F*01:01 complexes bound to peptides of non-canonical length. The models of pHLA-F complexes are based on the structure of HLA-F*01:01 (5KNM). (**a**) PBR of HLA-F*01:01 bound to the 8-meric peptide LILRWEQD (orange) from Dulberger et al. (**b**) HLA-F*01:01 bound to the 15-meric peptide AVFVDLEPTVIDEVR (red). (**c**) Structural model of the 19-meric peptide mVNPTVFFDIAVDGEPLGR (blue) bound to the HLA-F*01:01 PBR. In all structures the N-terminal site of the peptides is sticking out of the peptide binding region allowing high variability of the pHLA-F molecule

To understand the binding mode of HLA-F bound to the long peptides, we modeled selected peptides into the PBR of HLA-F*01:01. Consistent with the theory that the PBR of HLA-F is unique among the HLA-I molecules, all peptide analysis showed that the N-terminal site of the peptides is not anchored into the PBR. Representative peptides were docked into the PBR of HLA-F using Rosetta FlexPepDock and are illustrated in Fig. 3.

## Discussion

HLA-F belongs to the non-classical HLA-Ib molecules with a marginal polymorphic nature and very restricted tissue distribution. HLA-F is directly involved in immune functions during HIV-1 infection and confers a beneficial effect on disease outcome. Through interaction with the NK cell receptor KIR3DS1, antiviral downstream immune responses are initiated and lead to a delayed disease progression. On the surface of activated CD4^+^ T cells, HLA-F has been reported to be upregulated; however, the interaction between HLA-F and KIR3DS1 is diminished (Garcia-Beltran et al. 2016). The non-polymorphic nature of HLA-F suggests that this dynamic immune response is elicited through the presented peptides; the structure of HLA-F bound to a natural selected and presented peptide illustrated the variability of pHLA-F complexes (Dulberger et al. 2017).

In this study, we aimed to systematically analyze the peptide variability of the predominant allelic variant HLA-F*01:01 and the peptide-mediated structural alteration of pHLA-F*01:01 complexes. Although it was reported that HLA-F is an unstable molecule with low frequency on the cell surface (Lee et al. 2010; Lee and Geraghty 2003; Wainwright et al. 2000), we were able to recover stable pHLA-F complexes using soluble HLA technology (Kunze-Schumacher et al. 2014). A total of 16 single peptides with 8 to 21 AAs in length could be identified; the presentation of non-canonical peptides by HLA-Ib molecules has been observed and described previously (Kraemer et al. 2015; Celik et al. 2018a; Dulberger et al. 2017). It has been described that HLA-Ia molecules also bind peptides with extraordinary length from 11 to 25 AAs (Hassan et al. 2015; Bell et al. 2009); those peptides bulge out of the peptide binding groove and extend the accessible surface for the corresponding immune receptor (Rist et al. 2013; Bade-Doding et al. 2011). The structure of HLA-F bound to peptides with > 10 AAs in length suggests that HLA-F restricted peptides are only C-terminally anchored, while the N-terminus protrudes out sustainably of the peptide binding groove. In the present study, we could identify Arg to anchor the peptides C-terminally. Furthermore, we could not identify a N-terminal anchor AA. These finding corresponds to previous structural analysis of HLA-F by Dulberger et al. mentioning that the PBR of HLA-F forms one open-ended groove (Dulberger et al. 2017). HLA-F*01:01 bound peptides do not form a constrained structure like extended peptides in other HLA molecules normally do (Hassan et al. 2015). However, since HLA-F bound peptides protrude N-terminally out of the PBR, the accessible surface of pHLA-F molecules for the corresponding immune receptor changes and KIR3DS1 might bind in an unconventional way.

Comparing this observation with peptide characteristics and the expression features of other HLA-Ib molecules, it becomes obvious that HLA-Ib molecules are pathogen-driven and tissue specifically expressed and those features reflect on the i) peptide repertoire, where peptides under pathogenic conditions are preferentially available and selected and the ii) peptide anchor motifs (Kraemer et al. 2015; Celik et al. 2018a). The binding of different peptides changes the landscape of HLA-Ia and -Ib molecules leading to a different interaction with receptors of the immune system. Most of the described receptors can be found on NK cells and are upregulated when HLA-Ia molecule expression is downregulated. Pathogen invasion triggers an exquisite immune balance of evasion through downregulation of class Ia molecules mediating NK cell activation and upregulating Ib molecules mediating NK cell inhibition. The recruitment of distinct immune cells during certain phases of infectious episodes promotes immune evasion of foreign or infected tissues. Therefore, the knowledge of the accessible HLA surface for a receptor is indispensable to understand effector cell recruitment; a fundamental step to achieve this objective is the analyses of the HLA specific peptide repertoire. Several studies indicate HLA allele-specific peptide selection. From the whole proteomic content, each HLA allele presents exclusively a minor selection. The main expression site of HLA-F are HIV-infected cells, it is likely that peptide selection for HLA-F presentation might be more specific for the proteomic source and/or undergo competition before presentation. Interestingly, we could determine peptides mainly derived from cytoplasmatic or nucleoplasmatic origin. Most of the HIV protein interaction partners are also located in the nucleoplasm (Ptak et al. 2008). Therefore, we analyzed HIV-1/human protein interactions and compare these interacting proteins with the source proteins of the recovered HLA-F restricted peptides. The analysis revealed that almost all HLA-F restricted peptides are derived from proteins that are involved in immune interaction with HIV proteins.

That HLA-F*01:01 selects self-peptides from healthy cells that are derived from HIV interaction partners suggests that peptide selection is source specific and not only sequence specific. The presentation of non-canonical peptides that exhibit an unusual binding mode affects stability and half-life times of protein complexes. pHLA-F complexes might be only for a distinct time frame available for the survey of NK cells and KIR3DS1 recognition.

The results presented in this study will contribute to the understanding of HLA-F function.

## Electronic supplementary material

ESM 1 (PDF 78.7 kb)

ESM 2 (PDF 218 kb)

ESM 3 (DOCX 104 kb)
