# Supplementary material for: HLA-F*01:01 presents peptides with N-terminal flexibility and a preferred length of 16 residues
Source: Immunogenetics. 2019 Apr 2;71(5):353–60. doi: 10.1007/s00251-019-01112-1 (PMC6525141; doi:10.1007/s00251-019-01112-1)
Supplement: Supplementary file 1 — (PDF 78.7 kb) [file 251_2019_1112_MOESM1_ESM.pdf]

Supplementary figure 1: Analysis of sHLA-F fractions after affinity chromatography

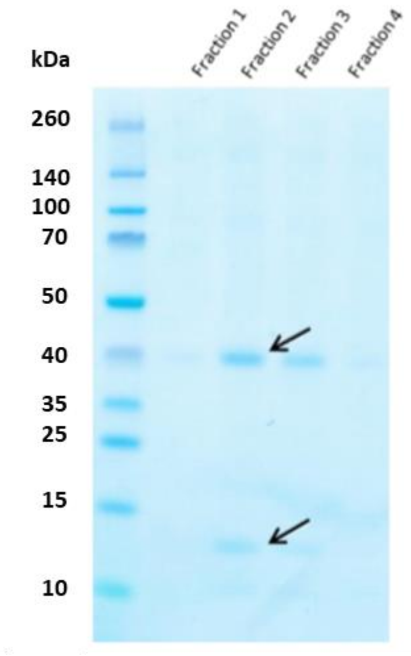

SDS-PAGE of sHLA-F*01:01; all samples were diluted 1:2; *Spectra Multicolor Broad Range* was used as maker; arrows indicate the position of HLA-F hc/V5-His6 (~39 kDa) and β2m (11,7 kDa).
