# Supplementary material for: HLA-F*01:01 presents peptides with N-terminal flexibility and a preferred length of 16 residues
Source: Immunogenetics. 2019 Apr 2;71(5):353–60. doi: 10.1007/s00251-019-01112-1 (PMC6525141; doi:10.1007/s00251-019-01112-1)
Supplement: Supplementary file 2 — (PDF 218 kb) [file 251_2019_1112_MOESM2_ESM.pdf]

Supplementary figure 2: Representative LC-MS spectrum of HLA-F peptides

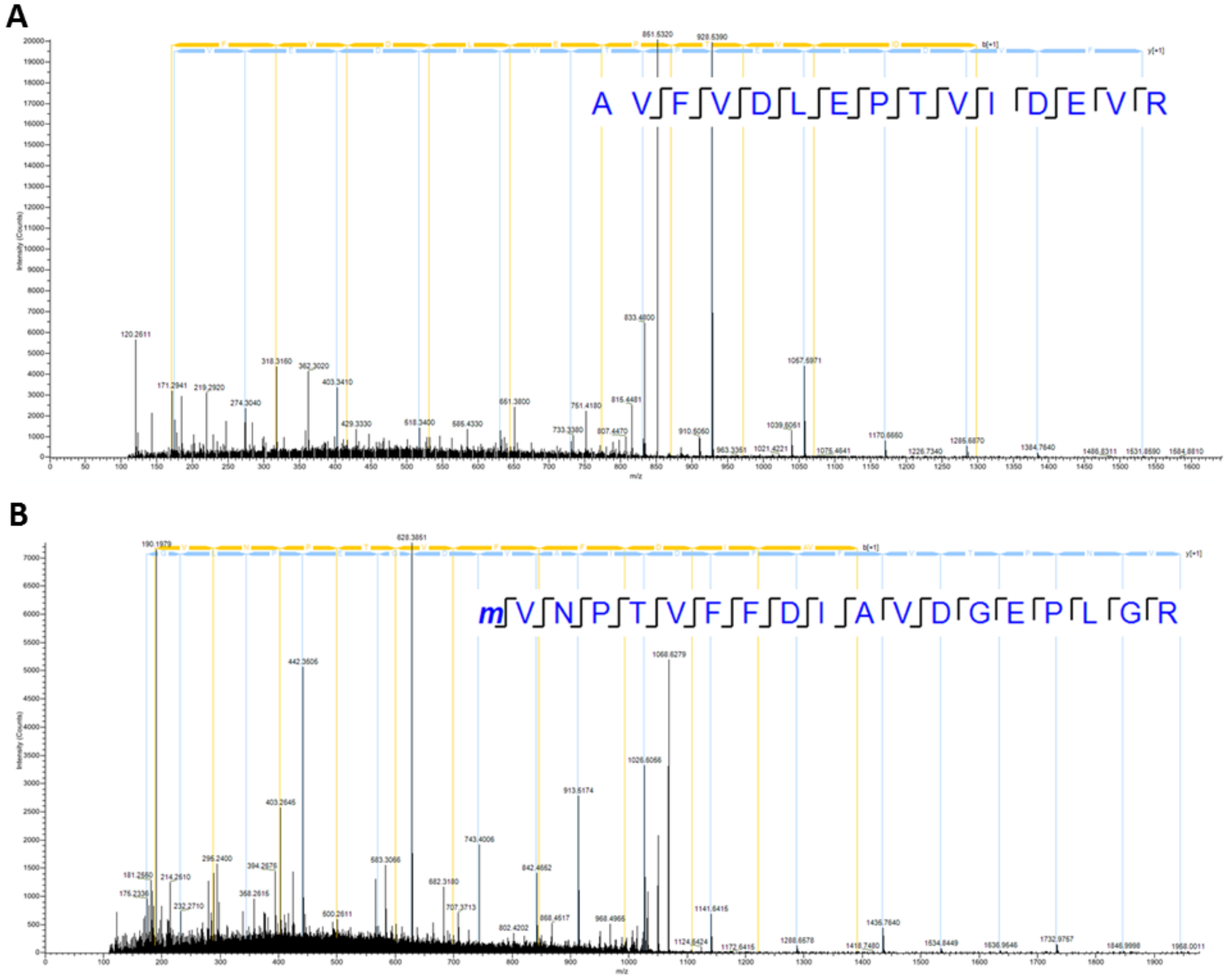

A) LC-MS spectrum of the peptide AVFVDLEPTVIDEVR. B) LC-MS spectrum of the peptide mVNPTVFFDIAVDGEPLGR. Coverage of y- ions (blue) and b- ions (yellow) allowed the identification of the peptides.
