# Supplementary material for: HLA-F*01:01 presents peptides with N-terminal flexibility and a preferred length of 16 residues
Source: Immunogenetics. 2019 Apr 2;71(5):353–60. doi: 10.1007/s00251-019-01112-1 (PMC6525141; doi:10.1007/s00251-019-01112-1)
Supplement: Supplementary file 3 — (DOCX 104 kb) [file 251_2019_1112_MOESM3_ESM.docx]

**Supplementary table 1: HLA-F restricted peptides are derived from proteins that interact with HIV-1 proteins**

| Protein name | Effect on HIV disease | HIV protein interaction | HIV gene | References |
| --- | --- | --- | --- | --- |
| proline rich coiled-coil 2B | N/A | N/A | N/A | N/A |
| glyceraldehyde-3-phosphate dehydrogenase | negative | Envelope surface glycoprotein gp120  Gag-Pol  [Nef](https://www.ncbi.nlm.nih.gov/protein/28872818)  Pr55  Rev  Tat  [Vpr](https://www.ncbi.nlm.nih.gov/protein/28872817) | *env*  *gag-pol*  *nef*  *gag*  *rev*  *tat*  *vpr* | [1]  [1, 2]  [1]  [2]  [3]  [4]  [5] |
| tubulin alpha-1B chain | beneficial | Envelope surface glycoprotein gp120  Envelope surface glycoprotein gp160, precursor  Pr55(Gag)  Rev  Tat  Integrase  [Retropepsin](https://www.ncbi.nlm.nih.gov/protein/25121906) | *env*  *env*  *gag*  *rev*  *tat*  *gag-pol*  *gag-pol* | [6, 7]  [8]  [8]  [3, 9]  [10]  [11]  [12] |
| heterogeneous nuclear ribonucleoprotein A1 | negative | Envelope surface glycoprotein gp120  Envelope surface glycoprotein gp160, precursor  Gag-pol  Nef  Pr55  Rev  Retropepsin | *env*  *env*  *gag-pol*  *nef*  *gag*  *rev*  *gag-pol* | [1]  [13]  [1]  [1]  [1, 13]  [3]  [12] |
| histamine receptor H3 subunit peptide 4 | N/A | N/A | N/A | N/A |
| annexin A1 | negative | Envelope surface glycoprotein gp120 | *env* | [14] |
| pyruvate kinase M | beneficial | Envelope surface glycoprotein gp120  Envelope surface glycoprotein gp160, precursor  Gag-pol  Nef  Pr55  Tat  VPR  Retropepsin | *env*  *env*  *gag-pol*  *nef*  *gag*  *tat*  *vpr*  *gag-pol* | [1]  [15]  [1]  [1]  [1]  [16, 17]  [18]  [12] |
| synovial sarcoma translocation gene on chromosome 18-like 1 | N/A | N/A | N/A | N/A |
| peptidyl-prolyl cis-trans isomerase A | beneficial | envelope surface glycoprotein gp120  [Nef](https://www.ncbi.nlm.nih.gov/protein/28872818)  Pr55  Vif  Vpr  Capsid  Integrase  Matrix  Nucleocapsid  p6  Retropepsin  Reverse transcriptase | *env*  *nef*  *gag*  *vif*  *vpr*  *gag*  *gag-pol*  *gag*  *gag*  *gag*  *gag-pol*  *gag-pol* | [19]  [20]  [21, 22]  [23]  [24]  [25, 26]  [27, 28]  [29, 30]  [31]  [32]  [12]  [33, 34] |
| beta-enolase isoform X1 | N/A | N/A | N/A | N/A |
| YWHAZ protein | beneficial | Tat  Vpr  Integrase | *tat*  *vpr*  *gag-pol* | [16]  [35, 36]  [15] |
| usherin | N/A | N/A | N/A | N/A |
| beta-actin | beneficial | Envelope surface glycoprotein gp120  Envelope surface glycoprotein gp160, precursor  Envelope transmembrane glycoprotein gp41  Gag-Pol  Nef  Pr55  Rev  Tat  VPR  Matrix  Nucleocapsid  Retropepsin  Reverse transcriptase | *env*  *env*  *env*  *gag-pol*  *nef*  *gag*  *rev*  *tat*  *vpr*  *gag*  *gag*  *gag-pol*  *gag-pol* | [37, 38]  [8, 39]  [40]  [1]  [41]  [42]  [43]  [44, 45]  [18]  [46]  [47]  [12, 48]  [49] |

No interactions have been reported for histamine receptor H3 subunit peptide 4; proline rich coiled-coil 2B; beta-enolase isoform X1; synovial sarcoma translocation gene on chromosom 18-like 1; usherin.

Supplementary references:

1. Milev, M.P., et al., *Characterization of staufen1 ribonucleoproteins by mass spectrometry and biochemical analyses reveal the presence of diverse host proteins associated with human immunodeficiency virus type 1.* Front Microbiol, 2012. **3**: p. 367.

2. Kishimoto, N., et al., *Glyceraldehyde 3-phosphate dehydrogenase negatively regulates human immunodeficiency virus type 1 infection.* Retrovirology, 2012. **9**: p. 107.

3. Naji, S., et al., *Host cell interactome of HIV-1 Rev includes RNA helicases involved in multiple facets of virus production.* Mol Cell Proteomics, 2012. **11**(4): p. M111 015313.

4. Lin, M.H., et al., *A HIV-1 Tat mutant protein disrupts HIV-1 Rev function by targeting the DEAD-box RNA helicase DDX1.* Retrovirology, 2014. **11**: p. 121.

5. Ferrucci, A., M.R. Nonnemacher, and B. Wigdahl, *Extracellular HIV-1 viral protein R affects astrocytic glyceraldehyde 3-phosphate dehydrogenase activity and neuronal survival.* J Neurovirol, 2013. **19**(3): p. 239-53.

6. Xu, Y., et al., *HIV-1-mediated apoptosis of neuronal cells: Proximal molecular mechanisms of HIV-1-induced encephalopathy.* Proc Natl Acad Sci U S A, 2004. **101**(18): p. 7070-5.

7. Valenzuela-Fernandez, A., et al., *Histone deacetylase 6 regulates human immunodeficiency virus type 1 infection.* Mol Biol Cell, 2005. **16**(11): p. 5445-54.

8. Jolly, C., I. Mitar, and Q.J. Sattentau, *Requirement for an intact T-cell actin and tubulin cytoskeleton for efficient assembly and spread of human immunodeficiency virus type 1.* J Virol, 2007. **81**(11): p. 5547-60.

9. Watts, N.R., et al., *HIV-1 rev depolymerizes microtubules to form stable bilayered rings.* J Cell Biol, 2000. **150**(2): p. 349-60.

10. Zhang, L., et al., *Modulation of the stability and activities of HIV-1 Tat by its ubiquitination and carboxyl-terminal region.* Cell Biosci, 2014. **4**(1): p. 61.

11. Yamamoto, S.P., et al., *Huwe1, a novel cellular interactor of Gag-Pol through integrase binding, negatively influences HIV-1 infectivity.* Microbes Infect, 2011. **13**(4): p. 339-49.

12. Impens, F., et al., *A catalogue of putative HIV-1 protease host cell substrates.* Biol Chem, 2012. **393**(9): p. 915-31.

13. Lund, N., et al., *Differential effects of hnRNP D/AUF1 isoforms on HIV-1 gene expression.* Nucleic Acids Res, 2012. **40**(8): p. 3663-75.

14. Molina, L., et al., *Proteomic analysis of the cellular responses induced in uninfected immune cells by cell-expressed X4 HIV-1 envelope.* Proteomics, 2007. **7**(17): p. 3116-30.

15. Jager, S., et al., *Global landscape of HIV-human protein complexes.* Nature, 2011. **481**(7381): p. 365-70.

16. Lopez-Huertas, M.R., et al., *The presence of HIV-1 Tat protein second exon delays fas protein-mediated apoptosis in CD4+ T lymphocytes: a potential mechanism for persistent viral production.* J Biol Chem, 2013. **288**(11): p. 7626-44.

17. Jarboui, M.A., et al., *Nucleolar protein trafficking in response to HIV-1 Tat: rewiring the nucleolus.* PLoS One, 2012. **7**(11): p. e48702.

18. Barrero, C.A., et al., *HIV-1 Vpr modulates macrophage metabolic pathways: a SILAC-based quantitative analysis.* PLoS One, 2013. **8**(7): p. e68376.

19. Saphire, A.C., M.D. Bobardt, and P.A. Gallay, *Host cyclophilin A mediates HIV-1 attachment to target cells via heparans.* EMBO J, 1999. **18**(23): p. 6771-85.

20. Saxena, R., et al., *Proteomic profiling of SupT1 cells reveal modulation of host proteins by HIV-1 Nef variants.* PLoS One, 2015. **10**(4): p. e0122994.

21. Valiente-Echeverria, F., et al., *eEF2 and Ras-GAP SH3 domain-binding protein (G3BP1) modulate stress granule assembly during HIV-1 infection.* Nat Commun, 2014. **5**: p. 4819.

22. Streblow, D.N., et al., *Cyclophilin a modulates processing of human immunodeficiency virus type 1 p55Gag: mechanism for antiviral effects of cyclosporin A.* Virology, 1998. **245**(2): p. 197-202.

23. Kamada, K., et al., *Evasion from CypA- and APOBEC-mediated restrictions is insufficient for HIV-1 to efficiently grow in simian cells.* Microbes Infect, 2009. **11**(2): p. 164-71.

24. Solbak, S.M., et al., *The intriguing cyclophilin A-HIV-1 Vpr interaction: prolyl cis/trans isomerisation catalysis and specific binding.* BMC Struct Biol, 2010. **10**: p. 31.

25. Toth, F., et al., *Effect of internal cleavage site mutations in human immunodeficiency virus type 1 capsid protein on its structure and function.* FEBS Open Bio, 2016. **6**(8): p. 847-59.

26. Zhou, J., et al., *HIV-1 Resistance to the Capsid-Targeting Inhibitor PF74 Results in Altered Dependence on Host Factors Required for Virus Nuclear Entry.* J Virol, 2015. **89**(17): p. 9068-79.

27. Briones, M.S., C.W. Dobard, and S.A. Chow, *Role of human immunodeficiency virus type 1 integrase in uncoating of the viral core.* J Virol, 2010. **84**(10): p. 5181-90.

28. Briones, M.S. and S.A. Chow, *A new functional role of HIV-1 integrase during uncoating of the viral core.* Immunol Res, 2010. **48**(1-3): p. 14-26.

29. Bristow, R., et al., *Human cyclophilin has a significantly higher affinity for HIV-1 recombinant p55 than p24.* J Acquir Immune Defic Syndr Hum Retrovirol, 1999. **20**(4): p. 334-6.

30. Hammerschmid, F., et al., *Interactions of HIV-1 proteins with human T-cell cyclophilin A.* Ann N Y Acad Sci, 1996. **782**: p. 456-61.

31. Fricke, T., et al., *Human cytosolic extracts stabilize the HIV-1 core.* J Virol, 2013. **87**(19): p. 10587-97.

32. Solbak, S.M., et al., *HIV-1 p6-Another viral interaction partner to the host cellular protein cyclophilin A.* Biochim Biophys Acta, 2012. **1824**(4): p. 667-78.

33. De Iaco, A. and J. Luban, *Cyclophilin A promotes HIV-1 reverse transcription but its effect on transduction correlates best with its effect on nuclear entry of viral cDNA.* Retrovirology, 2014. **11**: p. 11.

34. Song, C. and C. Aiken, *Analysis of human cell heterokaryons demonstrates that target cell restriction of cyclosporine-resistant human immunodeficiency virus type 1 mutants is genetically dominant.* J Virol, 2007. **81**(21): p. 11946-56.

35. Planelles, V. and S. Benichou, *Vpr and its interactions with cellular proteins.* Curr Top Microbiol Immunol, 2009. **339**: p. 177-200.

36. Kino, T., et al., *Vpr protein of human immunodeficiency virus type 1 binds to 14-3-3 proteins and facilitates complex formation with Cdc25C: implications for cell cycle arrest.* J Virol, 2005. **79**(5): p. 2780-7.

37. Garcia-Exposito, L., et al., *Gelsolin activity controls efficient early HIV-1 infection.* Retrovirology, 2013. **10**: p. 39.

38. Anand, A.R., et al., *N-terminal Slit2 inhibits HIV-1 replication by regulating the actin cytoskeleton.* Retrovirology, 2013. **10**: p. 2.

39. Warrilow, D. and D. Harrich, *HIV-1 replication from after cell entry to the nuclear periphery.* Curr HIV Res, 2007. **5**(3): p. 293-9.

40. Huang, S.H., et al., *HIV-1 gp41 ectodomain enhances Cryptococcus neoformans binding to human brain microvascular endothelial cells via gp41 core-induced membrane activities.* Biochem J, 2011. **438**(3): p. 457-66.

41. Tan, R., et al., *Nef interaction with actin compromises human podocyte actin cytoskeletal integrity.* Exp Mol Pathol, 2013. **94**(1): p. 51-7.

42. Le Sage, V., et al., *Proteomic analysis of HIV-1 Gag interacting partners using proximity-dependent biotinylation.* Virol J, 2015. **12**: p. 138.

43. Kimura, T., et al., *A role for Rev in the association of HIV-1 gag mRNA with cytoskeletal beta-actin and viral protein expression.* Biochimie, 1996. **78**(11-12): p. 1075-80.

44. Raborn, E.S., et al., *Cannabinoid inhibits HIV-1 Tat-stimulated adhesion of human monocyte-like cells to extracellular matrix proteins.* Life Sci, 2014. **104**(1-2): p. 15-23.

45. Al Soraj, M., et al., *siRNA and pharmacological inhibition of endocytic pathways to characterize the differential role of macropinocytosis and the actin cytoskeleton on cellular uptake of dextran and cationic cell penetrating peptides octaarginine (R8) and HIV-Tat.* J Control Release, 2012. **161**(1): p. 132-41.

46. Hearps, A.C. and D.A. Jans, *Regulating the functions of the HIV-1 matrix protein.* AIDS Res Hum Retroviruses, 2007. **23**(3): p. 341-6.

47. Lyonnais, S., et al., *A protein ballet around the viral genome orchestrated by HIV-1 reverse transcriptase leads to an architectural switch: from nucleocapsid-condensed RNA to Vpr-bridged DNA.* Virus Res, 2013. **171**(2): p. 287-303.

48. Shoeman, R.L., et al., *Non-viral cellular substrates for human immunodeficiency virus type 1 protease.* FEBS Lett, 1991. **278**(2): p. 199-203.

49. Bukrinskaya, A., et al., *Establishment of a functional human immunodeficiency virus type 1 (HIV-1) reverse transcription complex involves the cytoskeleton.* J Exp Med, 1998. **188**(11): p. 2113-25.
